# Using Science-Driven Analog Research to Investigate Extravehicular Activity Science Operations Concepts and Capabilities for Human Planetary Exploration

**DOI:** 10.1089/ast.2018.1861

**Published:** 2019-03-06

**Authors:** Kara H. Beaton, Steven P. Chappell, Andrew F.J. Abercromby, Matthew J. Miller, Shannon E. Kobs Nawotniak, Allyson L. Brady, Adam H. Stevens, Samuel J. Payler, Scott S. Hughes, Darlene S.S. Lim

**Affiliations:** ^1^ KBRwyle, Houston, Texas.; ^2^ NASA Johnson Space Center, Houston, Texas.; ^3^ Jacobs Technology, Houston, Texas.; ^4^ Department of Geosciences, Idaho State University, Pocatello, Idaho.; ^5^ School of Geography and Earth Sciences, McMaster University, Hamilton, Canada.; ^6^ European Astronaut Centre, European Space Agency, Cologne, Germany.; ^7^ BAER Institute, Moffett Field, California.; ^8^ NASA Ames Research Center, Moffett Field, California.

**Keywords:** Extravehicular activity, Science operations, Extreme environments, Human spaceflight analog, Concepts of operations

## Abstract

Biologic Analog Science Associated with Lava Terrains (BASALT) is a science-driven exploration program seeking to determine the best tools, techniques, training requirements, and execution strategies for conducting Mars-relevant field science under spaceflight mission conditions. BASALT encompasses *Science*, *Science Operations*, and *Technology* objectives. This article outlines the BASALT Science Operations background, strategic research questions, study design, and a portion of the results from the second field test. BASALT field tests are used to iteratively develop, integrate, test, evaluate, and refine new concepts of operations (ConOps) and capabilities that enable efficient and productive science. This article highlights the ConOps investigated during BASALT in light of future planetary extravehicular activity (EVA), which will focus on scientific exploration and discovery, and serves as an introduction to integrating exploration flexibility with operational rigor, the value of tactical and strategic science planning and execution, and capabilities that enable and enhance future science EVA operations.

## 1. Introduction

The BASALT (Biologic Analog Science Associated with Lava Terrains) program includes *Science*, *Science Operations*, and *Technology* objectives that are being addressed through real (nonsimulated) biogeochemical fieldwork under simulated Mars mission constraints that are based on current architectural assumptions for future exploration missions (see Lim *et al.*, 2019, for an overview of the BASALT project). The BASALT Science program is investigating how microbial communities and habitability correlate with physical and geochemical characteristics of chemically altered basalt environments in Mars-analog terrestrial sites (Brady *et al.*, 2019; Hughes *et al.*, 2019; Lim *et al.*, 2019). The BASALT Science Operations and Technology programs are examining concepts of operations (ConOps) and capabilities that enable and enhance scientific return during human–robotic exploration under Mars mission constraints (Beaton *et al.*, 2019; Marquez *et al.*, 2019; Miller *et al.*, 2019; Seibert *et al.*, 2019; Norheim *et al.*, 2018).

The purpose of this article is to introduce the BASALT Science Operations program. Here, we present (1) an overview of how current (and former) extravehicular activity (EVA) operations will shift for future planetary exploration, (2) the previous analog work that informed the ConOps and capabilities selected for investigation during BASALT, (3) the Science Operations study design, and (4) a detailed description of field test execution. We also report a small subset of Science Operations results from the second field deployment, referred to as BASALT-2, related to EVA execution; the primary set of BASALT-2 Science Operations results are presented in the work of Beaton *et al.* (2019).

BASALT-2 took place in the Mauna Ulu region on the Big Island of Hawai'i in November 2016. This field test built on the lessons learned from BASALT-1, which was conducted at Craters of the Moon (COTM) National Monument and Preserve in Idaho in June 2016 (Beaton *et al.*, 2017), and it was the predecessor for BASALT-3, which took place in the Kilauea Iki and Keanakakoi regions of Hawai‘i in November 2017.

### 1.1. Challenges for future planetary EVA

Safe and effective EVA will be a critical component of any future human space exploration mission (Drake, 2009a, 2009b). EVA is the means by which astronauts explore and interact with their surroundings within the habitable environment of their spacesuit (McBarron, 1994). Since Gemini IV in 1965, NASA has incrementally advanced EVA through a series of flagship programs, including Gemini, Apollo, Skylab, the Space Shuttle, and the International Space Station (ISS). These programs have established EVA as a mission-critical capability with demonstrated success in spacecraft and payload construction, inspection, maintenance, and repair and in lunar surface exploration (Portree and Trevino, 1997).

As NASA and partner space agencies aim to someday land humans on the surface of Mars (ISECG, 2018), a host of new challenges to enable safe and productive EVA must be addressed. One significant factor is the transition from operating on engineered surfaces, such as for Shuttle and ISS EVA, to exploring unfamiliar “natural” terrains, including the moons and surface of Mars (Chappell *et al.*, 2013a, 2013b; Craig *et al.*, 2015; Gernhardt *et al.*, 2016). The scale, unpredictability, and physical hazards associated with natural environments will demand human and system capabilities well beyond prior experiences (Greeley, 1999; Rummel *et al.*, 2014). Of the 410 EVAs completed to date (as of December 2018), only nine have been conducted on planetary bodies with objectives emphasizing science (Apollo J-class missions 15, 16, and 17) (Packham and Stockton, 2016). Hence, there is limited spaceflight experience to inform the design and implementation of future planetary exploration of EVA (Miller *et al.*, 2016a, 2017; Greenlund *et al.*, 2017).

Planetary EVA operations should be designed and executed in a manner that takes full advantage of capabilities that are unique to human explorers in pursuit of scientific objectives (Hodges and Schmitt, 2011). Science objectives will impose new tasks and decision-making paradigms that differ significantly from those of past and current EVA. Traditional, engineering-based EVA tasks are more readily translatable to detailed, sequential procedures with obvious success criteria. Future EVA objectives will focus on scientific discovery and exploration, will be less tightly constrained (potentially with more undetermined success criteria), and will continually evolve with the acquisition of new information. As a consequence, planetary EVA operations may need to cope with a greater degree of flexibility than what has been traditionally allowed. Historically, flexibility has been minimized during the design of EVA plans because flexibility can lead to unpredictability, thereby increasing risk to the crew, vehicles, and the ability to complete objectives. Therefore, the effective integration of scientific flexibility with strict safety and operational considerations, including EVA life support, hardware, and vehicle systems, will be a key objective that must be addressed to ensure future mission success (Schmitt *et al.*, 2011).

Communication latencies and bandwidth limitations between Mars astronauts and Earth-based mission support add additional complications for EVA planning and execution. EVA operations to date have relied on near real-time communication between space and ground and have incorporated parallel mission control teams dedicated to timeline and life support system management, crew health and physiology, and resource utilization (Caldwell, 2000; Miller *et al.*, 2015, 2017). However, one-way light time (OWLT) communication delays between Earth and Mars range from 4 to 22 min depending on planetary alignment. Further, the level of interaction between astronauts and ground support will be affected by the bandwidth available, which may limit, for example, the transmission of video, high-resolution imagery, or large amounts of instrument data (Rader *et al.*, 2013). Hence, a new equilibrium will need to be established between the astronauts and Earth support to ensure adequate allocation of resources, authority, and responsibility (Love and Reagan, 2013; Fischer and Mosier, 2014; Kintz *et al.*, 2016).

Although communication latencies create new challenges for space-to-ground (SG) interactions, they do not preclude the ability for remote, Earth-based experts to add scientific value to EVA. The high-profile, high-risk, high-reward nature of conducting scientific exploration on Mars, coupled with the fact that no amount of crew training can replace the expertise available on Earth to cover the breadth, depth, scope, and complexity of Mars science objectives, means that incorporating as much knowledge as possible from the Earth will be highly desirable. Hence, future EVA timeline (the temporal order of EVA phases and tasks) and traverse (the spatial routes through areas of interest that the crew will explore) design may benefit from incorporating varying amounts of ground assimilation time (GAT; the time that Earth-based scientists and operators have to make decisions affecting crewmembers' subsequent actions without the crew incurring idle [*i.e.*, nonproductive] time).

Unlike most field expeditions on the Earth, Mars science objectives and execution will be much more heavily constrained by operational considerations, such as the increased risks to crewmembers associated with conducting EVA for longer periods of time or performing excessive numbers of EVA. Other important factors include transport costs, EVA suit consumables costs, and the potential for increased cross-contamination if multiple visits to sites of high biological sensitivity are planned. Each of these aspects will need to be balanced to achieve the best science at the lowest cost and risk.

Finally, there are technological challenges of building new systems to support planetary science EVA operations. Existing EVA support hardware and software have largely remained unchanged since they were first designed in the 1960s and 1970s (Frank *et al.*, 2008; Smith and Korsmeyer, 2011). Modern-day hardware and automated software technologies provide a wide variety of perceived utilities that could be leveraged in the EVA community, much like the fly-by-wire systems now found on modern-day aircraft. The EVA community faces the challenge of appropriately selecting from numerous technologies and systems that may support novel ConOps for future EVAs on such places as Mars.

The extravehicular (EV) crew (*i.e.*, the crewmembers outside in spacesuits) and intravehicular (IV) crew (*i.e.*, the crewmembers supporting the EV crew from within a local vehicle or habitat) exploring Mars could enhance their EVA science and operations through the integration of capabilities (*i.e.*, technologies and systems), such as position mapping, navigation aids, handheld science instruments, and the transmission and receipt of audio, video, and text data. Likewise, Earth-based mission support personnel could benefit from not only monitoring transmissions from the EV and IV crew, but also incorporating tools that allow them to rapidly ingest and analyze the incoming data and to interact with the crew, thereby providing meaningful input to the explorers. A challenge lies in determining not only what future work practices and responsibilities will be needed for future domain operators, but also which capabilities are most enabling to these operators for different Mars operational concepts, whose missions are constrained by communication latencies and bandwidth restrictions.

In summary, each of these challenges for future exploration EVA (*i.e.*, evolving from deterministic procedures to flexible exploration, incorporating scientific expertise on Earth while subject to communication latencies and bandwidth limitations, and leveraging new support capabilities to enable the future EVA crew and ground work practices) will need to be addressed before sending humans to Mars. Strategies to do so have been investigated in various Earth-based spaceflight analogs (Section 1.2.) and form the foundation of the BASALT Science Operations research objectives and study design.

### 1.2. Earth-based operational field tests as high-fidelity analogs for planetary EVA

As described by Lim *et al.* (2019), there are a variety of terrestrial research analogs that mimic key aspects of spaceflight and provide meaningful platforms to prototype and iteratively test and evaluate the ConOps and capabilities needed for future exploration missions. ConOps are the instantiation of operational design elements that guide the organization and flow of personnel, communications, hardware, software, and data products involved in a mission concept. Analog missions increase the maturity of ConOps through formalized testing, data collection and analysis, and validation. They also provide substantial cost-saving benefits by uncovering technical and operational deficiencies in a terrestrial environment before flight and promote innovative collaborations between government agencies, private industry, and academia that can share resources and reduce operational costs (Reagan *et al.*, 2012; Rader *et al.*, 2013). By building on previous analog tests and aggregating the results of multiple analog projects, spaceflight operators, scientists, and researchers are better able to prepare for the challenges of future exploration missions.

Over the past several decades, NASA, along with its international, commercial, and academic partners, has led numerous analog field tests in Arctic, Antarctic, underwater, desert, cave, and volcanic environments (Lim *et al.*, 2019). The Exploration Analog and Mission Development (EAMD) team at NASA Johnson Space Center (JSC) has led science operations research for many of these analogs, including the Desert Research And Technology Studies (DRATS), Pavilion Lake Research Project (PLRP), NASA Extreme Environment Mission Operations (NEEMO), and the BASALT program. This section highlights some of these previous analog missions and associated capabilities (italicized below) that were used to inform the ConOps, capabilities, and Science Operations objectives investigated during BASALT.

During the DRATS project, several years of high-fidelity field testing were conducted at Black Point Lava Flow, north of Flagstaff, Arizona. From 2008 to 2010, during NASA's Constellation Program, DRATS testing focused on systematic evaluation of hardware and ConOps for enabling simulated scientific exploration of the lunar surface with near real-time support from one or more teams of scientists on the Earth (Abercromby *et al.*, 2010, 2012, 2013b). This testing informed the development and evaluation of a *mobile instrument platform* (MIP) to assist crewmembers with science and science operations. A MIP can range from a small unmanned robot to a large unpressurized or pressurized human-rated vehicle (Burridge *et al.*, 2003), but in all cases, it consists of a mobility system combined with a minimum set of capabilities that are relevant to science and science operations. DRATS testing baselined and assessed several enabling features for a MIP, including (1) a mobility system capable of following or transporting crew, tools, scientific instruments, and samples within 100 m of the science locations of interest; (2) position-tagged, high-resolution imagery from mast-mounted, remote-controlled panoramic and pan-tilt-zoom situational awareness (SA) cameras; and (3) communication and navigation systems to enable connectivity between EV crew, IV crew, and Earth-based scientists and operators (Abercromby *et al.*, 2013b).

Although communication latency was not a consideration for the 2008–2010 DRATS' focus on lunar exploration, important lessons were learned with respect to the management of collaborative team members in space and on the Earth. Testing, as well as premission traverse planning (Hörz *et al.*, 2013; Skinner and Fortezzo, 2013), informed the organization of flight controllers and science support personnel into specific console positions with detailed procedures and flight rule documents.

During DRATS 2010, science experts were further organized into *Tactical* and *Strategic Working Groups*, each with defined roles and responsibilities (Eppler *et al.*, 2013). The tactical science operations team oversaw intra-EVA crew activities, whereas the strategic science operations team evaluated the overall progress of the mission science objectives and re-planned future EVA traverses as needed during post-EVA planning shifts. Both the tactical and strategic working groups were found to be critical parts of the science process and necessary to achieving the desired mission science return.

After the Constellation Program cancellation and a new direction to explore more distant destinations (Drake, 2009a; Craig *et al.*, 2015), the 2011 DRATS field test (Abercromby *et al.*, 2013a) and a multi-week Research And Technology Studies (RATS) test conducted at JSC in 2012 (Abercromby *et al.*, 2013c) focused on adapting lunar ConOps and capabilities to mission architectures that included four to six crewmembers traveling to a deep space asteroid and interacting with ground support teams across longer communication latencies. Various strategies for *distributing EV and IV crewmember roles* were evaluated and compared, spreading three or four astronauts across a deep space habitat and multi-mission space exploration vehicle. These studies demonstrated a preference for two IV crewmembers (versus only one) when there were two EV crewmembers, as two IV crew enabled more effective management of both the operations and science aspects of the EVA (Abercromby *et al.*, 2013c).

One DRATS test condition evaluated the effect of significantly reduced SG bandwidth, with results indicating only a modest impact on science operations; the low bandwidth condition (1.5 Mb/s downlink from space to ground) was found to be borderline acceptable, whereas the high bandwidth condition (6 Mb/s downlink) was found to be acceptable with the differences in the ratings attributed to a decrease in science value due to changes in video frame rate and high-resolution panoramic images downlink rates (Abercromby *et al.*, 2013b); see the work of Beaton *et al.* (2019) for definitions of acceptability.

*Communication coverage maps* were found to be important for EVA traverse planning and execution to ensure that the EV crew did not lose communication with the IV crew or the Earth at critical moments. Multiple communication capabilities, including *multi-way audio* and *multi-way texting* among the EV crew, IV crew, and ground support and *video transmission* from EV crew-mounted chest cameras, were compared and evaluated across several communication latencies and found to provide varying levels of enhancement. Other capabilities, such as *laser-based feature pointers* (used by EV crew to direct attention to specific terrain features of interest), were also evaluated and found to be useful.

In parallel to the DRATS and RATS investigations, complementary shirtsleeve and spacesuit testing was conducted at NASA JSC to investigate human-suit interactions, suit and vehicle consumables (*e.g.*, breathing gases, power, and water), and differences in task performance and duration between suited and unsuited operations. These results served as the basis of understanding EVA resource requirements that could, for example, be used to maximize scientific exploration range while reducing fatigue and injury risk to the crew (Scheuring *et al.*, 2009; Chappell *et al.*, 2010, 2017; Norcross *et al.*, 2010a, 2010b).

Although it is recognized that suited operations impact EVA timelines and traverses, these studies demonstrated that conducting both unsuited and suited tests provides a more complete picture of EVA operations. For example, unsuited field testing provides a natural environment for investigating science tasks and the features and functions of capabilities needed to support EVA objectives without overly burdening field operations with unrealistic representations of planetary EVA suits (*e.g.*, lightweight mockups or prototypes not designed for 1g environments) (Abercromby *et al.*, 2013b). Parallel suited testing in gravity-offload environments provides realistic measurements of biomechanical adaptations, modifications of energy expenditures, and task timing information due to suited operations (Valle *et al*. (2011).

The PLRP was initiated to investigate real (nonsimulated) scientific exploration under simulated deep space exploration ConOps. Over the 10-year project (2004–2014), a combination of SCUBA divers, remotely operated vehicles, autonomous underwater vehicles, and piloted DeepWorker 2000 submersibles (Nuytco Research Ltd.) conducted series of scientific research dives (*i.e.*, simulating EVA activities using EV and IV crewmembers) to examine microbialite formation and growth (Lim *et al.*, 2010, 2011). The EVA crew were guided by topside “Earth-based” science support teams over communication latencies ranging from 50 s to 5 min. A number of new science operations concepts and capabilities were developed and tested at PLRP, which laid the foundation for future analog work during NEEMO and BASALT analog missions.

Through PLRP field tests it was determined that special considerations for EVA timeline design were needed to best enable intra-EVA interactions between the in-space crewmembers and Earth-based science experts operating under communication latencies. For example, conducting multiple, sequential *presampling surveys* before any sampling provided time for Earth-based scientists to examine incoming presampling data and provide expert sampling recommendations without the EVA crew incurring idle time. The use of *physical feature-of-interest markers* enabled the EVA crewmembers to unambiguously communicate with the scientists across latency about specific targets of interest.

The concept of a *scientific dynamic leaderboard* in which the scientists continuously ranked candidate sampling targets in order of scientific priority was developed and implemented; sharing this leaderboard regularly with the crew ensured that the crew had the most up-to-date information should they encounter communication dropouts, consumables constraints, and/or other operational factors that meant sample collection needed to happen sooner than expected or without “final” priorities being received from the scientists.

*High-resolution still imagery* was found to be more useful scientifically than video footage streaming from EV crewmember helmet cameras, although these video feeds were important for operational SA. Finally, PLRP reaffirmed, under real scientific exploration, the importance of *two IV crewmembers*, with one primarily focused on operations and the other on science, as well as the need for enabling dynamic flexibility within the EVA to best enable science (Miller *et al.*, 2016b).

NEEMO missions 18–21 (2014–2016) further evaluated and refined the exploration ConOps and capabilities identified during DRATS and PLRP. During these missions, NEEMO crewmembers lived inside the Aquarius underwater habitat (Shepard *et al.*, 1996) for up to two weeks and conducted daily simulated EVAs in the sand channels and coral reef surrounding Aquarius. NEEMO crewmembers communicated across 5- or 15-min OWLT delays with a topside Mission Control Center (MCC) and science support team (Chappell *et al.*, 2016). NEEMO missions 18 and 19 focused on refinements to the timeline design evaluated during PLRP to investigate the ideal number of scientific areas of interest to visit per EVA and the durations needed for presampling surveys to maximize available GAT for Mars-relevant communication latencies.

NEEMO missions 20 and 21 applied these lessons learned to real scientific exploration and sampling of the reef. During these missions, oral descriptions, footage from EV crewmembers' helmet-mounted video cameras, and scientific instrument data were collected during the presampling phases and used by the scientists to recommend targets for sampling. The PLRP dynamic leaderboard concept for organizing sampling priorities and providing regular guidance to the crew was further matured and found to be essential. PLRP physical feature markers, now referred to as *candidate sample markers*, were upgraded to incorporate scale bars, color bars, and North arrows; as in previous analog testing, the markers were found to be critical for unambiguously communicating information relevant to targets of interest between space and ground.

Cursory capabilities for *image annotation* of screen captures from EV crewmembers' helmet-mounted video feeds by members of the science team were explored and found to be promising for relaying specific information about the surrounding terrain (*e.g.*, where to gather scientific instrument data and where to sample) back to the crew. In addition, the concept of a *tactical EVA timeline management tool* was developed and evaluated. The tool successfully provided the IV crew with the ability to manage the EVA by tracking the EV crew's progress against the planned timeline, projecting deviations in the start and end times of future tasks based on the actual durations of prior tasks, and notifying the crew of key milestones when they could expect guiding input from the science backroom team.

### 1.3. Concepts of operations and baseline architecture

The selection and design of the ConOps investigated during BASALT was guided by current NASA administration goals and previous analog field experiences. Two important underlying assumptions framed this ConOps. First, for future exploration destinations such as Mars, robotic precursor missions (orbital and/or surface) will have collected imagery and precursor data to plan science exploration EVA traverses to be conducted by human crew (NASA, 1972; Hodges and Schmitt, 2011). On execution of the EVAs by the crew, additional levels of information (*i.e.*, beyond the precursor data) will be obtained through contextual and presampling surveys of each location of interest that may modify traverse plans, science tasks, and/or science priorities (Chappell *et al.*, 2016; Miller *et al.*, 2016b; Beaton *et al.*, 2017).

Second, as noted in Section 1.1, a higher level of scientific expertise will exist on the Earth than with the crew (Eppler *et al.*, 2013). Although crewmembers will be highly trained, they will not be the sole experts in all relevant disciplines for Mars science objectives. Hence, strategic integration of Earth-based expertise is assumed to enhance scientific return (Yingst *et al.*, 2011). This integration can occur within EVAs (***intra***-EVA SG interactions) and between EVAs (***inter***-EVA SG interactions). ConOps that incorporate intra-EVA interactions are important and advantageous when science objectives drive the exploration of as many different regions of interest as possible (which will be operationally constrained by a fixed amount of transport and EVA consumables in a given amount of time) or when science objectives limit the number of visits to a particular site (*e.g.*, to reduce the potential for biological cross-contamination).

The ConOps investigated during BASALT focuses specifically on EVAs that integrate tactical, intra-EVA expertise from Earth while promoting scientific productivity, operational efficiency and safety, and minimal crew idle time.^*^ Note that although substantial science data analysis and EVA re-planning occurred daily between EVAs, this strategic element of mission operations was not a focus for evaluation of the BASALT project. The tactical, intra-EVA SG interaction ConOps directly addresses the presumption that latency and bandwidth constraints associated with deep space missions preclude meaningful “real-time” communication with Earth and, hence, demand nearly complete crew autonomy (Pohlkamp *et al.*, 2015; Frank *et al.*, 2016).

From an EVA perspective, this translates into an assumption that intra-EVA SG interactions, especially in the form of scientific guidance, become more challenging as communication latency increases and bandwidth allowance decreases. This challenge can be addressed through deliberate traverse and timeline design and management, as described in the paragraph below and in Beaton *et al*., 2019. Further, this ConOps necessitates that capabilities on both the space and ground sides be designed for maximum efficiency and effectiveness since EVAs are inherently short in duration; this includes efficiencies in data transmission, receipt, analysis and interpretation, etc.

For intra-EVA SG interactions, science exploration traverses can be designed without crew incurring idle time by prospectively defining which tasks can be completed independent of Earth input and which tasks are either dependent on or could substantially benefit from Earth input (Chappell *et al.*, 2016). For example, a dependent task pair could consist of a presampling survey (*e.g.*, contextual audio descriptions, still imagery, and video footage of candidate sampling locations identified by the EV crew) and a corresponding sampling task at a particular location of interest. The EV crew could complete a presampling survey and send that data to the Mission Support Center (MSC), which the MSC could then analyze and interpret to guide sampling recommendations. While the MSC is receiving the data across latency and formulating their recommendations, the EV crew could complete a second presampling survey or a separate independent (*i.e.*, stand-alone) task. With sufficient understanding of EVA task dependencies, task durations, communication delays, and necessary GAT, timelines and traverses can be created that allow for Earth input on many tasks while minimizing or avoiding crew idle time and promoting flexibility in the EVA objectives based on new information arriving from the EVA crew (Chappell *et al.*, 2016; Miller *et al.*, 2016b).

The ConOps studied during BASALT consists of a defined set of personnel with specific roles and responsibilities, relevant environments and facilities within which the work is conducted, and a set of capabilities that facilitate the work. In-simulation (in-sim) personnel include two EV crewmembers in the field completing science exploration and sampling tasks and two IV crewmembers working from an IV workstation (inside a simulated rover or habitat) supporting the EV crew. The IV crew communicate with an Earth-based MSC that provides scientific expertise and operational guidance across communication latency and bandwidth limitations (Lim *et al.*, 2019; Fig. 2).

The use of the word “support” in MSC rather than “control” (as in the MCC that has been used for low Earth orbit operations) reflects the advisory role that the MSC assumes when working under communication latency. Table 1 describes the roles and responsibilities of the primary in-sim EVA personnel.

**Table 1.** Biologic Analog Science Associated with Lava Terrains In-Simulation Personnel Roles and Responsibilities

Mars crew	Two EV crewmembers: in the field cooperatively completing science tasks; EV1 (EV operations lead) leads timeline management, traverse navigation, and other operational tasks, whereas EV2 (EV science lead) leads the science execution.
	Two IV crewmembers: inside an IV workstation guiding the EVAs; IV1 (IV operations lead) primarily interacts with the EV crew and MSC (via CAPCOM) on operational tasks, timelines, constraints, and procedures, whereas IV2 (IV science lead) primarily interacts with the EV crew and MSC (via SCICOM) on science tasks, priorities, and recommendations.
Earth MSC	Flight Director: has authority over all operational recommendations from the MSC.
	CAPCOM: communicates with IV1 on operational tasks, timeline, constraints, and procedures.
	SCICOM: communicates with IV2 on science tasks, priorities, and recommendations; tracks EVA timeline and keeps the science team apprised of critical bingo times based on current communication latency.
	EVA Planner: monitors and updates timeline based on EV crew progress; assists SCICOM with tracking critical bingo times; and works with SCICOM to relay messages to IV
	Science Team Lead: has authority over all scientific recommendations from the MSC; leads the science team in providing tactical feedback to EV/IV crew.
	Biology Lead: provides feedback regarding features that may have an impact on habitability and/or the microbial community based on incoming data from EV crew; provides input for dynamic leaderboard; and records observational notes and contamination concerns.
	Geology Lead: provides feedback on significant geological features based on incoming data from EV crew; provides input for dynamic leaderboard; and records observational notes.
	Instrument Lead: examines scientific instrument data from the field and offers additional interpretation based on analysis; provides input for dynamic leaderboard based on instrument data; and records instrument data notes.
	Imagery Lead: notifies the science team when new still imagery arrives from the field; carefully examines the details of the incoming imagery; and tags the images with relevant contextual information.
	Leaderboard Lead: records the science priorities, alternatives, and rationale based on science team discussions in a dynamic leaderboard.
	Tactical Awareness Management Lead: carefully tracks precisely where the EV crew are in the EVA timeline and relays this information.
	Science Team Members: science experts that work with the leads to tactically and strategically plan and guide EVA execution.

EV, extravehicular; EVA, extravehicular activity; IV, intravehicular; MSC, Mission Support Center; CAPCOM, capsule communicator; SCICOM, science communicator.

A series of capabilities facilitate communication and data transmission between the EV crew, IV crew, and MSC and enable the scientific exploration and sampling to be conducted (Lim *et al.*, 2019; Figs. 2–5). These include:

1. Real-time (between EV and IV crew) and delayed (between the EVA crew and MSC) voice data.
2. Real-time and delayed text messaging.
3. Real-time and delayed transmission of video data from EV crew chest-mounted video cameras and an SA camera.
4. Real-time and delayed transmission of high-resolution still imagery from EV crew handheld point-and-shoot cameras.
5. Real-time and delayed transmission of scientific data from EV crew hand-held instruments [including a Fourier transform infrared spectrometer, visible-near infrared spectrometer, portable X-ray fluorescence spectrometer, and forward-looking infrared camera (Sehlke *et al.*, 2019)].
6. Science presampling and sampling tools, including candidate sample location markers and sterile sampling tools (Stevens *et al.*, 2019).
7. Real-time and delayed transmission of EV crew GPS position data.
8. A MIP consisting of a simulated rover mast-mounted SA camera, high-resolution panorama camera, mobile-automated light detection and ranging (LiDAR) instrument, and communication network relay (Miller *et al.*, 2019).
9. EV crew graphical wrist displays for viewing GPS position tracks, text messages, annotated images, and video feeds streaming from the chest-mounted cameras (Miller *et al.*, 2019).
10. *Minerva*—an integrated science support tool comprising the Exploration Ground Data System (xGDS) software package (Deans *et al.*, 2017), Playbook timeline management tool (Marquez *et al.*, 2013), and SEXTANT traverse optimization tool (Norheim *et al.*, 2018). Minerva enables the (a) creation, modification, and display of EVA traverses; (b) creation, modification, and display of EVA timelines, including a tactical EVA timeline management tool to automatically track overall EVA times (*e.g.*, time elapsed and time remaining) and critical task times, and provides a sophisticated science operations management platform and repository for scientific data, imagery, and field notes (Marquez *et al.,* 2019).

Two primary voice communication loops are employed during the EVAs: SG-1, across which the EV and IV crew converse in real-time, and SG-2, across which the IV crew and the MSC communicate across latency. The SG-1 loop is transmitted to the MSC across delay so that the MSC can hear the EV-IV crew conversations. EV crewmembers do not listen to the SG-2 loop. Real-time and delayed text messaging and annotated image sharing is provided by the Playbook Mission Log (Marquez *et al.*, 2019). Still images, EV crew chest camera video streams, and EV crew GPS position tracks are relayed to the IV crewmembers in real-time and to the MSC (as bandwidth allows) across delay through the xGDS software tool.

All data are automatically archived in the xGDS database and semantically linked to the current EVA. Relevant tags and descriptive notes can be appended to each still image by the IV crewmembers or members of the MSC to enable search functionality used during sampling prioritization, post-EVA analysis, and future EVA planning (Marquez *et al.*, 2019).

### 1.4. Deriving actionable results from analog testing

For analog missions to provide actionable results for follow-on testing, design recommendations, functional requirements definition, and, ultimately, future spaceflight operations, rigorous assessment methodologies are needed to systematically evaluate the ConOps and capabilities under investigation. In 2008, the EAMD team was chartered by the NASA Directorate Integration Office to oversee and conduct numerous spaceflight analog mission evaluations (including at DRATS, PLRP, NEEMO, and BASALT) utilizing a consistent set of operational methods and metrics to enable the iterative development, testing, analysis, and validation of evolving exploration architectures. This approach ensures that the required level of rigor and consistency is applied before, during, and after the operational field tests so that the data collected remain relevant to NASA's strategic architecture and technology development goals and provide data-driven, actionable recommendations. Key points include:

1. The definition of strategic questions and rationale behind each.
2. An understanding of how results will be used (*e.g.*, to inform requirements, design recommendations, etc.) and corresponding justification(s).
3. The development of functional requirements, objectives, and hypotheses (*i.e.*, expected outcomes) related to the questions being tested.
4. The prospective definition of metrics that will be used to assess the objectives and accept/reject the hypotheses, including levels of significance.
5. The development of a study design that incorporates all necessary tasks to address the questions and objectives and a plan to collect the quantitative and qualitative data.
6. The documentation of assumptions.
7. The selection of test subjects that are representative of the target population (*e.g.*, flown astronauts) and the provision of sufficient training so that the subjects understand the objectives and methods for collecting their input.
8. The execution of the study design with adequate fidelity of the operational environment and inclusion of the relevant technologies to address the questions at hand.
9. The use of test subject consensus results to form a single set of data that reflect the agreed-on results of any subjective input provided.
10. The mapping of results to specific, actionable hardware, software, and/or procedural recommendations.

This approach has been employed at BASALT and is described briefly next and in detail in Beaton *et al.* (2019).

## 2. Methods

### 2.1. BASALT science operations strategic questions and study design

The BASALT Science Operations team is investigating five primary research questions, which are addressed through the implementation, testing, and evaluation conducted at each field test. These questions are based on current NASA administration architectural objectives and the results and lessons learned from prior analog tests to fill strategic knowledge gaps and build on previous accomplishments (Johnson and Buffington, 2017; Blanco and Aitchison, 2018).

The primary distinction between BASALT and previous analog projects is that the BASALT ConOps and capabilities assessments have been designed for evaluation during terrestrial biogeochemical fieldwork that directly links to astrobiological and geological plans for future Mars missions (Hughes *et al.*, 2019; NRC, 2007; Rummel *et al.*, 2014; MEPAG, 2015). Each research question focuses on conducting EVA operations in a manner that best enhances science. The BASALT Science Operations research questions are:

**Science Operations 1A:** Do the baseline Mars mission ConOps, systems, and communication protocols developed and tested during previous NASA analog tests work acceptably during real scientific field exploration? What improvements are desired, warranted, or required?

**Science Operations 1B:** Do these ConOps, systems, and communication protocols remain acceptable as communication latency increases from 5- to 15-min OWLT? What improvements are desired, warranted, or required?

**Science Operations 2A:** Which capabilities enable and enhance Mars scientific exploration EVA?

**Science Operations 2B:** Do these capabilities remain enabling and enhancing as communication latency increases from 5- to 15-min OWLT?

**Science Operations 2C:** Do these capabilities remain enabling and enhancing as communication bandwidth allowances decrease?

These research questions were addressed through a variety of objective and subjective assessments, which are outlined in Section 2.6.

The BASALT-2 field test consisted of ten simulated EVAs in which scientifically significant samples of basalt were extracted from “Mars” (*i.e.*, Mauna Ulu region, Hawai‘i). The EVAs were designed and conducted under two different communication latencies and two different bandwidth conditions:

- Latency: 5- and 15-min OWLT, which fall within the 4–22-min OWLT delays experienced between Mars and the Earth.
- Bandwidth: low = 0.512 Mb/s uplink and 1.54 Mb/s downlink (representing a conservative and lower-cost flight data rate), and high = 5.0 Mb/s uplink and 10.0 Mb/s downlink (representing an upgraded capability that would require additional infrastructure and technology development) (NRC, 2012; NASEM, 2016).

These communication delays and bandwidth conditions formed four communication study conditions: (1) 5-min latency/high bandwidth, (2) 5-min latency/low bandwidth, (3) 15-min latency/high bandwidth, and (4) 15-min latency/low bandwidth. All simulated EVAs were conducted under one of these four conditions. For BASALT-2, two EVAs were planned per study condition with two additional reserve EVAs for contingencies, such as inclement weather or technical difficulties. Premission estimates of data product sizes were calculated and post-EVA network analytics were completed to ensure that bandwidth traffic stayed within the study condition limits.

Three EVA teams were established based on the key roles described in Table 1, including three pairs of EV and IV crewmembers, three Science team leads, three science communicators (SCICOMs), and two sets of the other MSC operators and scientists. The personnel assigned to these teams came from the BASALT team of investigators, and all have extensive experience in science and/or operations through other field science and analog work. Teams were counterbalanced across the four communication study conditions.

### 2.2. EVA traverse and timeline design

Ten baseline EVAs were planned for BASALT-2. Precursor data included Google Earth^®^ imagery at a resolution of 0.15 m/pix, multispectral imagery at ∼2 m/pix, and digital elevation models at 10 m/pix. Scientists used these data to identify candidate locations of interest, referred to as “EVA stations”, relevant to their science research objectives (Brady *et al.*, 2019). EVA stations were grouped and organized into planned traverses, which included the routes between stations. Each EVA included three stations. Each station was ∼10 m in diameter, but station boundaries were adjusted if needed by the EV crew during EVA execution to facilitate meeting scientific objectives.

**FIG. 1.**
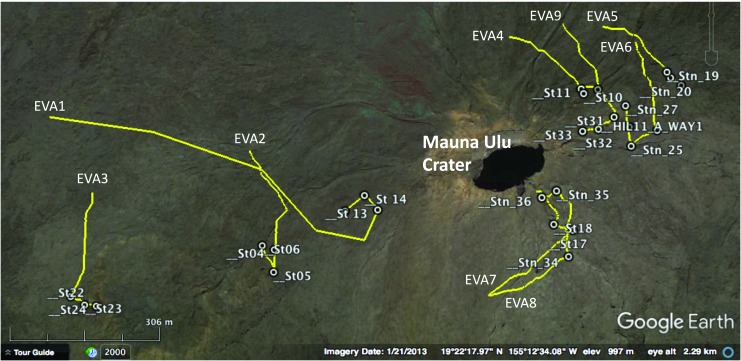
BASALT-2 planned EVA traverses. BASALT, Biologic Analog Science Associated with Lava Terrains; EVA, extravehicular activity.

As the field test progressed and new scientific information was gleaned from each EVA, traverse plans for subsequent EVAs were updated (including adjustments to science objectives, station locations, station boundaries, and routes between stations) to best meet the overall BASALT-2 science goals. In the end, BASALT-2 consisted of nine completed EVAs, whose planned traverses are depicted in Figure 1.

Each EVA consisted of five phases: station approach, station contextual survey, sample location search, presampling, and sampling. Timelines were strategically designed to minimize the potential for crew idle time by delineating EVA tasks that could be conducted independent of Earth input and tasks that either were dependent on or could substantially benefit from Earth input. Because three stations were visited during each EVA, the MSC was able to consider data from at least two stations before their first transmission of presampling recommendations was needed (regardless of whether the EVA was being conducted under 5- or 15-min OWLT communication latency). A representative EVA timeline is provided in Table 2. The following section describes the detailed tasks associated with each EVA phase.

**Table 2.** Representative Biologic Analog Science Associated with Lava Terrains-2 Planned Extravehicular Activity Timeline

*Task no.*	*Planned duration (h:min)*	*Planned start (h:min, PET)*	*Planned end (h:min, PET)*	*Task*
1	00:15	00:00	00:15	Station A Approach
2	00:05	00:15	00:20	Station A Contextual Survey
3	00:30	00:20	00:50	Station A Candidate Sample Location Search
4	00:15	00:50	01:05	Station B Approach
5	00:05	01:05	01:10	Station B Contextual Survey
6	00:30	01:10	01:40	Station B Candidate Sample Location Search
7	00:15	01:40	01:55	Station C Approach
8	00:05	01:55	02:00	Station C Contextual Survey
9	00:30	02:00	02:30	Station C Candidate Sample Location Search
10	01:00	02:30	03:30	Presampling Survey
11	00:30	03:30	04:00	Sampling at Sample Location 1
12	00:30	04:00	04:30	Sampling at Sample Location 2

PET, phase elapsed time (0:00 represents the start of the EVA).

### 2.3. Detailed EVA tasks by EVA phase and EVA role

At the beginning of each EVA, the EV crew provided a brief local report, which included the current time, wind speed and direction, percentage cloud cover, sun angle, temperature, and amount of precipitation. During the station approach phase, the EV crew described the surrounding terrain and recorded still imagery and video along the traverse, noting important features that characterize the area and that relate to that day's science objectives. The EV crew also looked for, described, and imaged targets of opportunity that might be relevant to other science objectives and be worth visiting later in that day's EVA or returning to on a separate EVA (see Brady *et al.*, 2019 for further description on targets of opportunity). The station approach phase ended when the EV crew arrived at the perimeter of the station.

On arrival at the station perimeter, the EV crew conducted a contextual survey, in which they (1) positioned the SA camera with guidance from the IV crew; (2) gave a verbal contextual report that included the orientation, shape, general condition, and color of the station, details regarding the presence of water, fluids, and biomass within the station, and any additional information relevant to that EVA's scientific objectives; and (3) recorded relevant still imagery and video footage. After the contextual survey, the EV crew conducted a candidate sample location search where they searched for, labeled with candidate sample markers, verbally described, and imaged candidate samples of basalt that met that EVA's science objectives (Stevens *et al.*, 2019).

A station approach, contextual survey, and candidate sample location search was conducted sequentially for each of the three stations. The EV crew then conducted a single presampling survey. The presampling survey incorporated follow-on exploration of high-priority candidate samples from all three stations that were down-selected by the MSC. The presampling survey involved the collection of additional close-up and contextual imagery and scientific instrument data that provided information about the mineralogical and geochemical composition of the candidates (Sehlke *et al.*, 2019).

During the sampling phase, the EV crew extracted samples based on guidance from the MSC. Samples were collected as a suite of seven replicates: one large replicate and several smaller rock chips for geochemistry and petrographic analyses, three small rocks for microbiology culturing and DNA extraction, two large rocks for organic geochemistry analysis, and one large rock as an archival hand sample (Brady *et al.*, 2019). Due to temperature sensitivity of microbial communities and organic compounds of interest, the samples collected for microbiology and organic geochemistry were immediately transferred to cold/frozen storage as required after leaving the field.

During each EVA phase, the IV crewmembers assisted the EV crew through the timeline tasks via audio communication in real-time and conversed primarily via text messages and recorded field notes with the MSC across delay. As described in Table 1, IV (IV1, IV2) and EV (EV1, EV2) crewmembers had similar but distinct roles and tasks to accomplish within an EVA. Both IV1 and IV2 monitored the mobile SA camera and EV crew video feeds streaming from the field. IV1 focused on the operational aspects of the EVA, whereas IV2 focused on the detailed science.

Specifically, IV1 (1) interacted with a tactical EVA management tool (a timeline spreadsheet that enabled the crew to monitor planned versus actual task start times, end times, and durations and to project future task start times based on how far ahead or behind the EV crew were from the planned timeline) and reported relevant timing information to the EV crew, (2) tracked GPS positions of the EV crew relative to the planned traverses and provided heading and distance information to the EV crew on request, (3) posted operationally relevant information to the Mission Log, (4) verified incoming still imagery and added tags and notes to each image within xGDS, and (5) monitored and responded to simulated EVA telemetry (including spacesuit consumable) data (Miller *et al.*, 2017).

The primary role of IV2 was to ensure that the scientific objectives of the EVA were met. Responsibilities included (1) distilling and communicating presampling and sampling priorities between the EV crew and MSC, (2) clarifying scientific queries between the MSC and EV2, and (3) providing both general and specific scientific guidance while observing the EVA in real-time (Kobs Nawotniak *et al.*, 2019).

Throughout the EVA, the MSC monitored and reviewed incoming data from the field across delay, recorded additional field notes in Minerva (Marquez *et al.*, 2019), and provided recommendations for presampling and sampling based on their collective expertise. The MSC used dynamic priority ranking lists (*i.e.*, dynamic leaderboards) to track and rank candidate samples relative to one another and against the science objectives for the current EVA and overall mission (Stevens *et al.*, 2019). The dynamic leaderboards were built from integrating and interpreting the incoming verbal descriptions, still imagery, video footage, and instrument data from the field, and they consisted of (1) priority rankings of the candidates under consideration (*i.e.*, numerical rankings starting with 1 up through the number of candidates), (2) relative priority ranking information that expressed how much greater priorities were over one another (*e.g.*, often expressed as priority 1 >> priority 2, or priority 1 ≅ priority 2), and (3) rationales associated with each priority ranking.

Updates to the presampling and sampling dynamic leaderboards were relayed regularly to the IV crew via the Mission Log, who could then discuss these rankings with the EV crew at an appropriate time. The use of these leaderboards enabled the crew to track the dynamic nature of the MSC recommendations and helped minimize crew idle time. The use of dynamic leaderboards is explored further in the work of Stevens *et al.* (2019).

### 2.4. Out-of-simulation support

Each EVA was supported by a network of out-of-simulation (x-sim) support personnel in the field and in the MSC. These individuals coordinated the start and stop of the EVAs, were on standby to assist with communication network troubleshooting, and took the place of mission critical capabilities (or aspects of capabilities) whose hardware and/or software designs were outside the scope of the BASALT project itself. For example, x-sim personnel replaced some of the MIP capabilities, including manually maneuvering the SA camera in the field and acting as containment units to carry sampling tools and extracted samples. Training was implemented to ensure in-sim and x-sim personnel maintained adequate simulation fidelity throughout all aspects of the EVA; detailed simulation quality results are presented in the work of Beaton *et al.* (2019).

The field support team (FST) assisted the EV crew in the field. The FST lead was responsible for coordinating all pre-, during-, and post-EVA x-sim activities. The instrument aide provided the EV crew with technical scientific instrument support, including carrying and handing over instruments to the crew at the appropriate times and troubleshooting malfunctions. The biology sterilization aide assisted the crew with critical biological sampling tasks to maintain sterilization. The field stenographer/runner recorded detailed field notes in the event of communication dropouts and assisted the FST lead as needed.

Two members of the BASALT communications and backpack team were responsible for maintaining communication coverage among the EV crew, IV crew, and the MSC and for providing EV informatics backpack (EVIB; Lim *et al.*, 2019, Figs. 4 and 5) troubleshooting support in the field; they were also responsible for moving the MIP communication relay and mobile SA camera to follow the EV crew.

Inside the MSC, the simulation coordinator (SIMCOORD) coordinated the start, end, and any pauses within the EVA with the FST lead. The MSC was further staffed with additional communications infrastructure support, Minerva technical support, a science operations stenographer (responsible for manually recording detailed EVA task timing data), and a science stenographer (responsible for manually recording detailed EV crew comments).

### 2.5. BASALT-2 flight rules

Shuttle and ISS EVA operations have generated a vast array of engineering and hardware flight rule constraints on system configurations (NASA, 1998). However, the shift in operational environments for future planetary EVA requires a shift in the style and content of flight rules for future mission operations. This includes promoting flexibility for scientific discovery and exploration and facilitating operations under substantial SG communication latencies and bandwidth limitations. BASALT has taken a first step in understanding and evaluating what flight rules for future planetary EVA operations might entail.

BASALT-2 flight rules were established to govern all aspects of field operations and to provide the operating guidelines with respect to safety, mission management and authority, EVA management and authority, troubleshooting, and ground rules (Table 3). The safety and the mission management and authority flight rules helped to ensure safety in the field and defined the conditions under which changes to EVA plans and personnel could be made. The EVA management and authority flight rules established the roles and responsibilities of the EVA crew and the MSC, emphasizing the “support” nature of the MSC rather than the classic “control” characteristic, which has been recommended when operating under communication latencies (Groemer *et al.*, 2014); these flight rules also limited total EVA duration and defined by who and when EVA extensions could be approved.

**Table 3.** Biologic Analog Science Associated with Lava Terrains-2 Flight Rules

*ID*	*Flight rule*
Safety	
S1	Any person may stop an activity (in-sim or x-sim) at any time for any reason to ensure safety of personnel and equipment and protection of the environment.
S2	EVAs shall end NLT 1600 HST.
Mission Management and Authority	
MM1	MMT has authority and responsibility for strategic (*e.g.*, EVA planning) decisions affecting scientific and/or exploration objectives. Strategic decisions affecting science and/or exploration objectives must be discussed with the MMT.
MM2	All EVA plans must be approved and finalized by the MMT at least 12 h before execution.
MM3	Minutes shall be recorded during all MMT meetings, including documentation of all decisions and plan changes.
MM4	Changes to EV, IV, Flight Director, and SIMCOORD shall be approved and finalized by the MMT at least 12 h before execution.
EVA Management and Authority	
EM1	The crew has authority and responsibility for tactical (*i.e.*, EVA execution) decisions:
	IV1: authority and responsibility for operational EVA decisions and tactics.
	EV2: authority and responsibility for scientific EVA decisions and tactics.
	MSC (including Flight Director, CAPCOM, SCICOM, and Science Team Members) are advisory only.
EM2	Flight Director has authority over all operational recommendations from the MSC.
EM3	Science Team Lead has authority over all scientific recommendations from the MSC.
EM4	CAPCOM and SCICOM are responsible for clear communication between the MSC and crew.
EM5	“In-sim” activities take priority over “x-sim” activities.
EM6	A single EVA shall not exceed 6 h.
EM7	EVAs shall be planned to fit a ≤5-h timeline plus 30-min margin.
EM8	Extensions up to 30 additional minutes (*i.e.*, beyond the 30-min margin called out in EM7 up to 6 h total PET) can be proposed NLT the planned end time of the EVA. Consent by all members is not required as long as the EV and IV crew, FST, and communications infrastructure team agree to the extension and the statement of extension is formally given to the MSC.
Troubleshooting	
T1	Minimum Acceptable Communication: Once started, simulations may continue under degraded communication conditions until indicated otherwise by the EV crew or FST (*e.g.*, EV crew determine they cannot execute EVA timeline without input from IV/MSC, or FST requires a system reboot to resolve system issues).
T2	Once an infrastructure reboot is determined essential, SIMCOORD shall coordinate with IV1 to schedule the reboot and notify FST. Once a reboot is completed, IV1 shall be notified by SIMCOORD and provide a 10-s countdown to resume the EVA.
T3	The dates and times during which in-sim EVAs are conducted without specific systems available shall be recorded by the science operations stenographer.
T4	Real-time position tracking and physiological monitoring are not required. EVA start times will not be delayed or interrupted to permit troubleshooting of physiological sensors.
T5	An established simulation not-to-exceed-end-time will be defined for each EVA (*e.g.*, not to exceed simulation time beyond 4:00 PM HST) by the MMT the day before operations. If troubleshooting may prohibit continuing of simulations for the day, an impromptu MMT meeting will take place to establish priorities for the remainder of the day.
T6	Troubleshooting in the field after an EVA shall not extend beyond the time required to get all personnel back to their vehicles before 1730 HST.
Ground rules	
GR1	A 20-m zone of exclusion will be implemented around the perimeter of the EV crew and EV support during the EVA to improve simulation fidelity and minimize area contamination.

CAPCOM, capsule communicator; EV, extravehicular; FST, field support team; HST, Hawai‘i standard time; IV, intravehicular; MMT, Mission Management Team; MSC, Mission Support Center; NLT, no-later-than; PET, phase-elapsed time; SCICOM, science communicator; SIMCOORD, simulation coordinator.

The troubleshooting flight rules defined the conditions under which the EVA could continue in the event of hardware, software, and/or communication malfunctions. Finally, the ground rules mandated an exclusion area around the EV crewmembers to facilitate quality in-sim operations. A mission management team (MMT) that included the leads of each BASALT sub-team (Science, Science Operations, Communications and EVIB, Minerva, and FST) was established to address concerns and necessary amendments to field operations during the field test and to ensure adherence to the flight rules.

### 2.6. Science operations data collection and analysis

Objective task performance data and subjective ratings of *acceptability*, *capability assessment*, and *simulation quality* (Abercromby *et al.*, 2013b) were collected during and after the EVAs to address the Science Operations research questions. Objective data included detailed EVA task timing information and SG interactions data, including (1) the number and type (*e.g.*, presampling, sampling) of interactions, (2) the timing of these interactions relative to the EVA timeline, (3) GAT available, (4) GAT utilized, and (5) EV crewmember idle time. The GAT available was defined by the EVA timeline and the communication latency under which the EVA was being conducted. The GAT utilized was derived from the timing of dynamic leaderboard updates sent to the crew.

Network usage data, which parsed total bandwidth usage by data type (including voice, text, still imagery, GPS position, etc.), were also collected (Miller *et al.*, 2019). Additional objective metrics included planned versus actual EVA phase durations, the number of candidate samples identified by the EV crewmembers, and traverse distances and durations by EVA phase. The objective results for BASALT-2 are presented in Section 3.

Field-tested subjective assessments were also incorporated to evaluate the ConOps and capabilities employed during the field deployments. These assessments included individual and consensus surveys of operational and scientific acceptability to evaluate the ConOps, communication protocols, and capabilities, and capability assessment ratings to describe how essential or enabling a particular capability was envisioned to be for future Mars exploration EVA. Simulation quality ratings were used to quantify the fidelity of the simulation implementation. The BASALT-2 acceptability, capability assessment, and simulation quality ratings are detailed in the work of Beaton *et al.* (2019).

## 3. Results

Before the start of the BASALT-2 field test, preliminary science objectives for each EVA were defined based on precursor data, which were developed into planned EVA traverses and timelines as described in Section 2.2. Hughes *et al.* (2019) details the field sites and baseline science objectives for the BASALT-2 EVAs. Once EVA execution commenced, science objectives were modified daily based on the cumulative results to date; as stated in Section 1.3, these strategic inter-EVA science re-planning efforts were not part of the BASALT Science Operations evaluations, but they are described in the work of Brady *et al.* (2019).

The BASALT-2 team deployed to the field several days in advance of the first planned EVA to set up and test the communication infrastructure, EVIBs, IV workstations, and MSC workstations (Miller *et al.*, 2019) and to provide additional time for the EV crewmembers to practice the EVA science tasks in representative terrain (Kobs Nawotniak *et al.*, 2019; Steven *et al.*, 2019). Three days of integrated testing and EVA dry runs were conducted, during which the three EVA teams rotated through their respective roles and tasks. The dry run days were followed by nine consecutive days of EVA, with one ∼4-h EVA completed per day. The four communication study conditions were counterbalanced across the nine EVA days.

The simulation quality for EVA 1 was rated unacceptable due to poor communication quality, which included inadequate communication coverage in the field and problems routing capabilities from the field to the MSC during critical portions of the EVA (Beaton et al. 2019). EVAs 2 through 9 were completed under sufficient simulation quality enabling meaningful evaluations of the ConOps, software systems, and communication protocols by using the acceptability and capability assessment ratings (Beaton *et al.*, 2019).

The following sections present the BASALT-2 Science Operations objective data.

### 3.1. EVA timeline execution

As described in Section 2.2, EVA timelines incorporated five phases to investigate the three science stations of interest. Timelines were strategically designed to facilitate SG interactions across latency and under bandwidth constraints to take advantage of MSC science expertise to better inform science priorities and tasks within the EVA. BASALT timeline durations were based on the time needed to accomplish the science objectives by using the tools and technologies designed for BASALT. Hence, BASALT timelines were not necessarily absolute representations of Mars EVA timelines, where analogous science would be conducted in spacesuits with flight-approved hardware and rovers for transportation and stowage. These timelines are, however, representative in the relative ratios of task duration to roundtrip communication latency, allowing time for iterative assimilation of data on the ground and feedback to the crew.

**FIG. 2.**
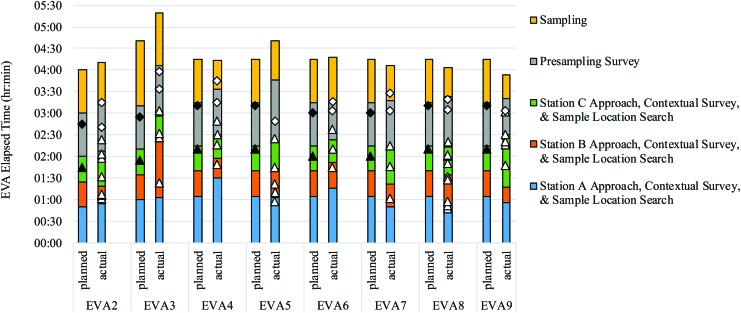
Planned and actual BASALT-2 EVA timelines in PET (h:min). Black triangles and diamonds represent the NLT deadlines for the MSC to send presampling and sampling guidance, respectively, to the EVA crew based on the communication latency present during that EVA. White triangles and diamonds show when the MSC sent presampling and sampling recommendations to the crew. MSC, Mission Support Center; NLT, no-later-than; PET, phase-elapsed time.

Figure 2 shows the planned versus actual EVA timelines for the eight EVAs completed under adequate simulation quality. In general, the crew tended to operate close to the planned timeline, although some tasks tended to exceed planned durations (see Section 3.3 for further discussion on actual EVA phase durations).

During each EVA, the MSC was faced with two critical no-later-than (NLT) “deadlines” in which MSC input regarding presampling and sampling recommendations had to be sent to the EVA crewmembers so that they would not incur idle time waiting on ground input. These NLT deadlines were based on the communication latency and assumed that the EV crewmembers would conduct the remaining EVA tasks according to the planned timeline. Hence, the MSC needed to send presampling and sampling guidelines NLT 5 or 15 min before the start of these phases. However, with the dynamic leaderboard approach, the MSC was encouraged to send multiple presampling and sampling priority rankings. Multiple updates to the crew in advance were important for minimizing idle time if the crew got ahead in the timeline and were critical in the event of communication network dropouts.

In Figure 2, the black triangles and diamonds on the planned timelines show the NLT deadlines for MSC presampling and sampling leaderboard input, respectively. The white triangles and diamonds on the actual timelines show when dynamic leaderboard presampling and sampling recommendations, respectively, were sent from the MSC to the crew. For each EVA, at least one presampling and one sampling leaderboard was sent to the crew, and hence no idle time was ever incurred by the crew due to waiting on presampling or sampling priorities from the MSC.

Typically, however, multiple presampling and sampling leaderboard updates were sent. As the EV crew continued to gather information in the field and the MSC assimilated these data across latency, the MSC regularly updated their leaderboards and sent several amendments to the crew. The crew understood the dynamic nature of these leaderboards, but because the MSC provided rationales and comments along with their priority rankings, the crew could use their best judgment as to how likely certain priorities might be to change.

Further discussion regarding the dynamic leaderboard approach, including how the leaderboards were managed in the MSC and when decisions were made to send inputs to the EVA crew, is provided in Stevens *et al.* (2019). The following section provides details on the planned versus actual science team leaderboard inputs and associated GATs. Section 4 describes the effectiveness and efficiency associated with these interactions and the overall success of the ConOps at achieving science results.

### 3.2. Science team leaderboard inputs and ground assimilation time

For the BASALT baseline ConOps to effectively enable the MSC to provide expert input to the EVA crew within the EVA, timelines had to be designed such that sufficient GAT was available to the MSC at the appropriate times. GAT was needed to ingest and interpret the incoming data from the field, discuss and rank the candidates, and communicate recommendations to the crew. Hence, as the EV crew identified, labeled, and described candidate samples, the MSC continuously assimilated the incoming information and dynamically prioritized the candidates for further presampling investigations or for sampling. The priorities and associated rationales were captured by the MSC Leaderboard Lead and communicated to the IV crew by the SCICOM using the Mission Log.

Table 4 shows a summary of the statistics related to the MSC-EVA crew interactions for presampling and sampling priorities across the EVAs. The table displays the number of candidates identified by the EV crew, the number of presampling and sampling leaderboard inputs sent by the MSC to the IV crew, presampling GAT, sampling GAT, and EV idle time due to waiting for MSC input. The number of candidates identified by the EV crew varied minimally across the EVAs (min = 8, max = 9). For all EVAs, multiple presampling leaderboard inputs were sent to the crew by the MSC and at least one (and in most cases at least two) sampling leaderboard input was sent.

**Table 4.** Dynamic Leaderboard Interactions Between the Mission Support Center and Extravehicular Activity Crew

	*EVA2*	*EVA3*	*EVA4*	*EVA5*	*EVA6*	*EVA7*	*EVA8*	*EVA9*
Number of candidates identified by EV	9	8	9	8	8	9	9	9
Number of leaderboard relays to IV								
Presampling	7	4	4	5	3	3	10	5
Sampling	2	2	2	1	2	2	2	1
Presampling GAT (h:min, PET)								
Planned for first NLT MSC input	1:45	1:55	2:10	2:10	2:00	2:00	2:10	2:10
Actual available for first NLT MSC input	1:53	2:41	2:26	2:15	2:08	1:55	2:11	2:26
Used for first MSC input	1:01	1:23	1:50	0:58	1:45	1:01	0:48	1:48
Sampling GAT (h:min, PET)								
Planned for first NLT MSC input	2:45	2:55	3:10	3:10	3:00	3:00	3:10	3:10
Actual available for first NLT MSC input	3:01	3:51	3:28	3:41	3:03	3:03	3:19	3:15
Used for first MSC input	2:40	3:34	3:14	2:50	3:03	3:03	3:04	3:04
EV idle time (h:min)	0:00	0:00	0:00	0:00	0:00	0:00	0:00	0:00

Times are presented as h:min.

EV, extravehicular; IV, intravehicular; GAT, ground assimilation time; PET, phase elapsed time; NLT, no-later-than; MSC, Mission Support Center.

GAT is presented in phase-elapsed time (PET), which is the total time since the start of the EVA. The GAT for both presampling and sampling is derived from the NLT times, that is, 5 or 15 min before the end of the Station C candidate sample location search. The *planned* GAT is based on the planned timeline. The *actual available* GAT is the planned GAT adjusted by the actual EVA phase durations up to (but not including) the Station C candidate sample location search. The *used* GAT is how much time the MSC actually used to send their first leaderboard recommendation to the crew.

The number of candidate samples identified by the EV crew varied little across the EVAs, whereas the number of presampling leaderboard inputs from MSC varied more (min = 3, max = 10). The difference in the number of presampling leaderboard inputs could be associated with the variation in the science objectives across the EVAs and with the number of candidates identified that met those objectives that needed to be considered (Steven *et al.*, 2019). In addition, there were differences in communication styles among the IV crew and members of the MSC across the three EVA teams, with some Science Team Leads preferring to update the IV crew with each leaderboard change, and others preferring to reserve updates until just before the actual NLT (Kobs Nawotniak *et al.*, 2019).

In general, it can be presumed that more GAT leads to better recommendations. However, establishing predefined roles, responsibilities, and communication channels within the MSC so that incoming data from the field are appropriately considered, expert science recommendations can be provided to the EVA crew across latency with relatively small amounts of GAT. These details are further expounded on in the works of Brady *et al.* (2019), Payler *et al.* (2019), and Stevens *et al.* (2019).

### 3.3. EVA traverse distance and duration

**FIG. 3.**
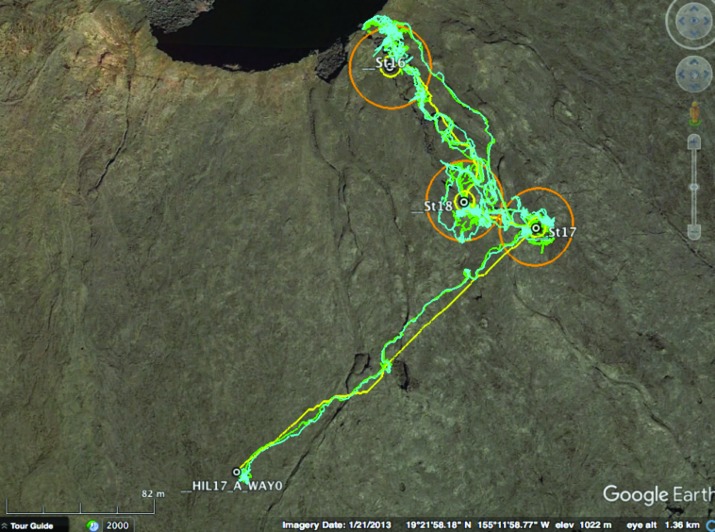
Planned (yellow line) and actual (green and cyan lines for EV1 and EV2, respectively) traverses for EVA 7. Yellow circle = 10-m diameter station boundary; orange circle = 40-m diameter approach circle around station.

As discussed in Section 1.2, EVA traverse distance and duration are important operational considerations, as they relate to EVA transport costs, consumables requirements, and general risks to the EV crewmembers (*e.g.*, fatigue, human-suit interactions injury risk, and an overall increased risk due to being out on EVA). Even though it is recognized that the traverse distances and durations during BASALT are not necessarily representative of operations in spacesuits and with rovers (see Sections 1.3 and 4.3 for further details), the data are valuable in a relative sense (*e.g.*, for relative comparisons of traverse distances and task durations across EVA phases) and for correlation with other objective data related to execution of the ConOps. Thus, actual EVA traverses were recorded for each of the nine BASALT-2 EVAs. As an example, Figure 3 depicts the actual traverses for the EV1 and EV2 crewmembers during EVA 7.

**FIG. 4.**
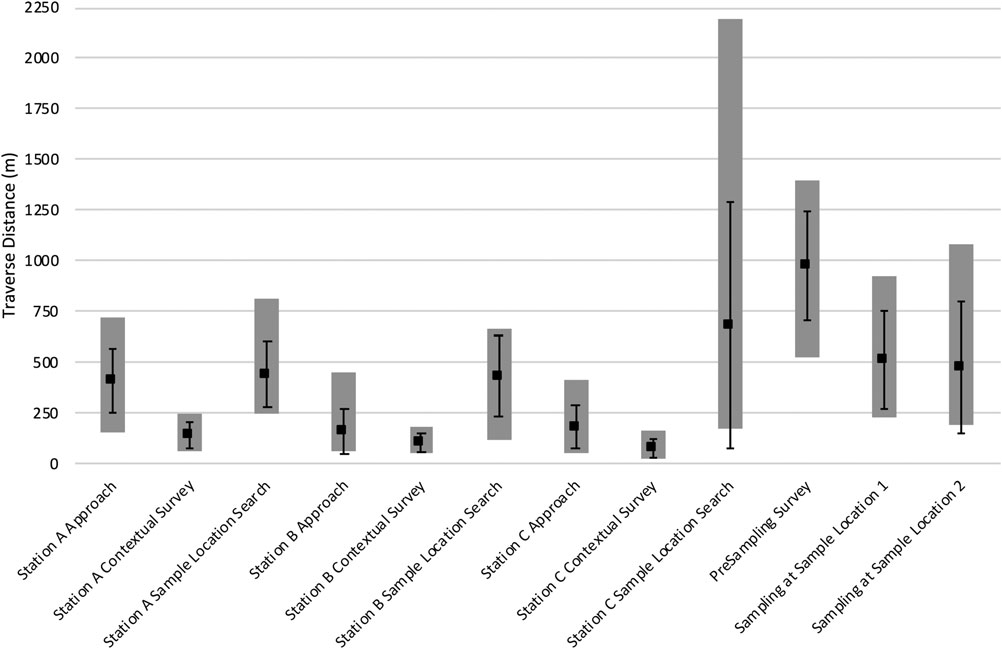
EVA mean traverse distance (m, ▪) for each EVA phase across all EVAs with adequate simulation quality; error bars represent standard deviation; gray columns represent range between minima and maxima.

**FIG. 5.**
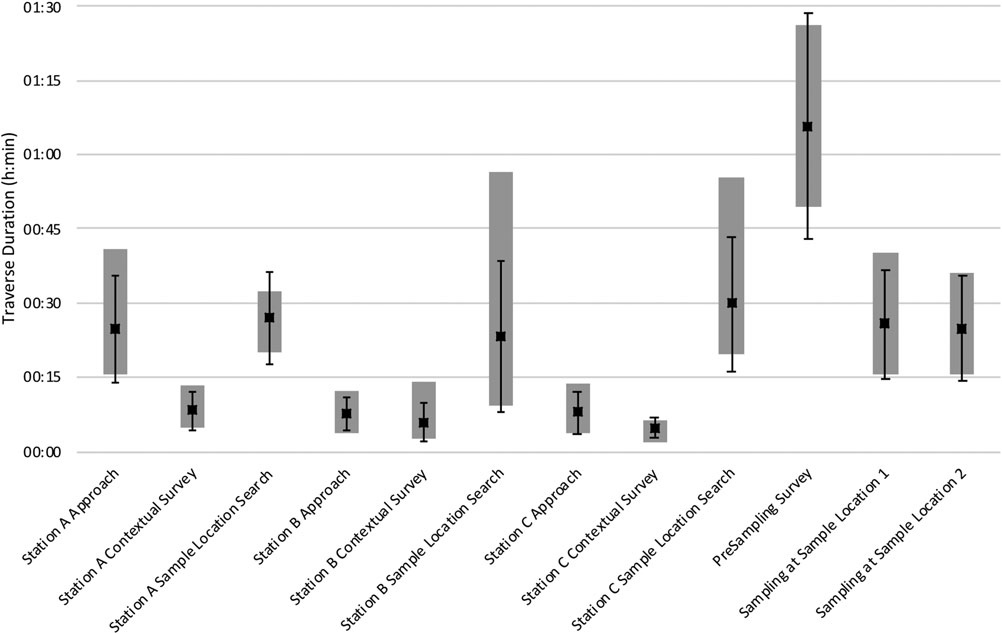
EVA mean traverse duration (h:min, ▪) for each EVA phase across all EVAs with adequate simulation quality; error bars represent standard deviation; gray columns represent range between minima and maxima.

Figures 4 and 5 present the EVA traverse distances and EVA phase durations, respectively, for each EVA phase. Each graph presents the mean distances or durations for all EVA phases across the eight EVAs with adequate simulation quality (EVAs 2–9); standard deviations, minima, and maxima are also included. In general, Station A approaches were associated with longer, more variable traverse distances and took longer to complete than Station B or C approaches. This was because the distances from the EVA start locations to the Station A perimeters were typically farther and more variable than the distances from Stations A to B and B to C.

The contextual surveys tended to take longer than the 5 min allocated in the planned EVA timelines. Although these EVA phases were intended to provide quick, high-level overviews of the station, the EV crew tended to give more detailed terrain descriptions. For the most part, the crew did not traverse much during the contextual surveys, although some crew occasionally circumnavigated the stations to facilitate more thorough descriptions; this was especially true when the stations incorporated terrain that was difficult to see around.

Traverse distances associated with Station C were the most variable. The EV crew or the MSC often adjusted the duration of this phase based on the quality of the candidates found at the previous stations. If adequate candidates to meet that EVA's objectives had been found at Stations A and B, then time spent searching for candidates at Station C was usually shortened; if inadequate candidates were found at Stations A and B, then time spent searching at Station C was typically extended. If insufficient candidates were found at any of the three stations, the EV crew were encouraged to extend the Station C candidate sample location search well outside of the Station C boundary.

The presampling survey distances traversed were dependent on three aspects: (1) the number of candidates selected for presampling, (2) the distances between the candidates, and (3) the priority order in which the candidates should be re-visited for presampling. In some cases, sample candidates were spread across all three stations and the priority rankings from the MSC leaderboards recommended that the crew traverse farther to ensure that the highest priorities were presampled first. In other instances, the candidates were closer together or the crew had more flexibility to decide which order to re-visit the priority candidates for presampling data collection. Finding the right balance between optimizing science objectives, which includes targeting the highest priorities first, while simultaneously minimizing EVA risks, including considerations for potential fatigue/injury or loss of SG communication, is a challenge for future exploration EVA that needs to be investigated further.

Sampling durations were dependent on the type of basalt being extracted and the number of replicates required to meet the science objectives. In general, the higher the level of alteration, the greater the friability and thus the easier the sampling; hence, highly altered basalt took less time to sample than unaltered basalt. In most instances, full suites of seven samples were collected from each candidate location, as described in the work of Brady *et al.* (2019) and Stevens *et al.* (2019). However, for some scientific objectives, only geological and archival samples were needed, in which case time was saved by not needing to extract as many samples or deal with sterile gloves, tools, and bags.

## 4. Discussion

### 4.1. Baseline concepts of operations and capabilities successes

An overarching goal of the BASALT project is to assess specific ConOps and capabilities for enabling Mars-relevant human scientific field exploration and discovery. The ConOps investigated during BASALT was evaluated for its ability to integrate Earth-based scientific (and operational) expertise *within an EVA* while subject to SG communication latencies and bandwidth limitations. Overall, this ConOps was successful in that scientists in the MSC were able to provide critical recommendations to the EV and IV crewmembers with the capabilities at hand, which enabled all science objectives for the BASALT-2 field test to be completed. Several important factors contributed to this success.

First, the science objectives were conducive to incorporating intra-EVA recommendations from the MSC. The primary aim of each EVA was for the EV crew to search for and collect samples of basalt that met certain levels of alteration and temperature (as described in Hughes *et al.*, 2019). The nature of the science objectives themselves, coupled with the high level of expertise within the MSC, enabled scientific recommendations to be provided to the crew in relatively short amounts of time.

Second, appropriate communication channels (including voice, text, video, still imagery, and science data) and baseline procedures for each were implemented to relay information from the field to the IV crew and the MSC and to accommodate SG interactions. Two SG loops were used to prioritize EV-IV conversations (SG-1) and to allow the crew and the MSC to interact (SG-2).

Third, individual roles and responsibilities within the EV crew, IV crew, and the MSC were defined to enhance effectiveness and efficiency; adequate training in these roles with the relevant capabilities was provided. The split responsibilities within the EVA crew, with EV1 and IV1 focusing primarily on operations and EV2 and IV2 focusing on science, enabled timeline management, navigation, and science to be conducted simultaneously. The IV crewmembers were the critical liaisons between the EV crew in the field and the MSC on Earth. The IV crew closely tracked the progress of the EV crew against the planned timeline and used their understanding of that day's science objectives to engage in an effective dialog with the MSC across latency regarding the candidate samples identified by the EV crew (Kobs Nawotniak *et al.*, 2019).

The division of labor within the MSC facilitated both tactical and strategic planning. Subteams (*e.g.*, geology, biology, imagery, etc.) coordinated among one another and reported to their subteam leads, who, in turn, reported to the overall Science Team Lead. CAPCOM (capsule communicator) and SCICOM clearly and concisely relayed critical information to the EVA crew at the appropriate times. The EVA planner tracked timeline progress and reminded the MSC of upcoming bingo times for relaying important recommendations (*e.g.*, NLT presampling and sampling leaderboards) to the EVA crew. Further details regarding individual roles and responsibilities, and their importance for tactical and strategic planning, are presented in the works of Kobs Nawotniak *et al.* (2019), Stevens *et al.* (2019), and Brady *et al.* (2019).

Fourth, pertinent capabilities were developed, incorporated, evaluated, and refined to enable the science and science operations. Critical capabilities included high-resolution still imagery, GPS position tracks, video feeds, and hand-held instruments that could detect mineralogical and geochemical composition of candidate samples (Sehlke *et al.*, 2019). The contextual and close-up still imagery collected by the EV crewmembers provided some of the most important science data collected during the EVA and served as the basis for many of the dynamic leaderboard rankings (Stevens *et al.*, 2019). Position tracks provided SA to the IV crew and MSC as to where the EV crew were in the terrain and where candidate samples were located relative to one another (Marquez *et al.*, 2019).

Video from the EV chest cameras and the mobile SA camera provided the IV crew (Kobs Nawotniak *et al.*, 2019) and the MSC with “real-time” tactical SA (Payler *et al.*, 2019) of crew locations and current EVA tasks. Stevens *et al.* (2019) describes the importance of still imagery combined with detailed audio descriptions as the most frequently used data products by the MSC in the candidate presampling prioritization process. Stevens *et al.* (2019) also explains the usefulness of transmitting scientific instrument data from the field for refining candidate sample priorities within a specific science objective.

Critical capabilities in the IV workstation and the MSC included the Minerva software tool (Marquez *et al.*, 2019) and the use of dynamic leaderboards (Steven *et al.*, 2019). Minerva temporally and geospatially synchronized all incoming data from the field and organized field notes captured by both the IV crewmembers and members of the MSC. The dynamic leaderboard approach minimized the risk of crew idle time and kept the EVA crew and MSC synchronized with respect to science priorities across latency.

Detailed post-EVA assessments of all baseline architecture capabilities implemented during BASALT-2 identified the level of mission enhancement afforded to the ConOps by their inclusion. These assessments also identified some additional Mars-forward capabilities. These results are presented in the work of Beaton *et al.* (2019).

Fifth, EVA timelines were strategically designed to facilitate flexibility for scientific exploration and GAT for the MSC to assimilate incoming data, discuss options, and provide recommendations to the crew. The sequencing of the EVA phases enabled the EVA crew to systematically provide additional information (beyond what was available in precursor datasets) about the stations and candidate sample locations to the MSC. This allowed the MSC to meaningfully and effectively integrate into the science being conducted by the EV crew. Although many factors affected the GAT available to the MSC, the MSC was successful in utilizing the training and tools (Marquez *et al.*, 2019) at their disposal such that no idle time was incurred waiting on MSC input. This was mainly achieved through the dynamic leaderboard approach, the first input of which was sent to the crew in advance of them starting the presampling and sampling phases.

As presented in Section 3, the actual EVA phase durations differed from the planned durations, which was expected given the inherent need to incorporate flexibility into scientific exploration plans. Additional factors that contributed to differences in planned versus actual EVA timelines and traverses include variability in the traversibility of the terrain (Norheim *et al.*, 2018), variations in the ability to find candidates meeting the science objectives within the planned stations (Brady *et al.*, 2019), the number of candidates selected for instrument evaluation during presampling surveys (Stevens *et al.*, 2019), the number of samples requested, and the difficulty of the sampling.

In summary, each BASALT field test was an iterative improvement over the previous one (Beaton *et al.*, 2017), with the first field test in Idaho in 2016 based on the composite lessons learned and recommendations from previous analog tests. Although the overall ConOps tested in BASALT-2 was successful for completing the science, important improvements and deficiencies associated with this ConOps and associated capabilities were identified during the subjective assessments (Beaton *et al*., 2019), which, in turn, informed the science operations objectives prioritized during BASALT-3.

### 4.2. Study limitations

Several important study limitations associated with the BASALT-2 Science Operations research need to be acknowledged. First, there were challenges with consistent execution of EVAs among the three different EVA teams. BASALT-2 was limited to thirteen consecutive field days: three for EVA training and integrated dry runs and ten for EVAs. Three different EVA teams were required to mitigate fatigue in the field and to incorporate sufficient numbers of individuals in the various roles to enable adequate consensus discussions and results for the acceptability and capability assessment ratings (Beaton *et al.*, 2019). Hence, balancing these factors led to some variations in task performance across the EVAs, including task durations and volume of SG interactions.

Next, there were challenges with successful operation of all desired capabilities across the EVAs. Occasional hardware troubleshooting, communication infrastructure instabilities, and unplanned drops in bandwidth availability occurred during some EVAs. In addition, there were several capabilities whose features and functions were unable to be consistently implemented in a flight-like manner. Notably, the EV crewmember's handheld camera did not always promptly transmit imagery, which led to either simulation troubleshooting pauses or delays in the MSC receiving the imagery. Since still imagery was critical for tactical science recommendations by the MSC, delays in imagery receipt had at least some minimal downstream effects on MSC decision making.

Futhermore, science instrument data were not able to be automatically transmitted across the communication network; instead, EV crew verbalized significant mineralogic results and took still images of spectral analysis screens provided by the instruments (Sehlke *et al.*, 2019). This led to nonflight-like inefficiencies for the crew and MSC. These limitations are represented in the simulation quality ratings presented in the work of Beaton *et al.* (2019).

Finally, the BASALT baseline architecture was intended for a MIP that could simulate a rover mast-camera system capable of collecting high-resolution panoramic images and mobile-automated LiDAR (Hurtado *et al.*, 2013). These capabilities were not able to be included in the BASALT-1 and BASALT-2 field tests. These capabilities provide science data to the MSC during the EVA, which has the potential to enhance both tactical and strategic EVA planning. BASALT-3 included these MIP capabilities, and the associated results will be presented elsewhere.

### 4.3. Forward work

The effects of different communication latencies, bandwidth limitations, available capabilities, the number and distribution of personnel, and other operational parameters investigated during previous analog studies led to the baseline ConOps and capabilities investigated during BASALT. BASALT field tests have provided an enhanced understanding of human planetary exploration science operations under the test conditions described earlier. However, logistical, temporal, and budgetary limitations precluded the ability to conduct individual field tests to investigate all possible combinations of ConOps, latencies, bandwidth conditions, science task types, etc. under the BASALT project. Hence, future work is warranted to augment the findings of BASALT.

The success of a unique exploratory EVA ConOps is science objective dependent. The BASALT-2 science objectives paired with the baseline ConOps and capabilities enabled the investigation of an Earth-based science team's ability to effectively and efficiently interact with crewmembers within an EVA. Although not all Mars science objectives (*e.g.*, longitudinal studies, studies that require testing during certain times of day) may be best served by the this ConOps, certainly some will be. For those that can, it is a significant advantage to know that expertise on the Earth can be used to inform decisions relatively quickly (*e.g.*, within an EVA), even under communication latencies or bandwidth constraints. Future work should seek to understand the wider variety of planned Mars science EVA objectives and associated SG timing requirements for their achievement.

Any factor that modifies task duration or phasing may result in sending more or less science-related data to the Earth (*i.e.*, due to increased or decreased GAT) than that which occurred during BASALT-2. Therefore, the overall effectiveness and efficiency of the ConOps under investigation may differ from what was found during BASALT-2. Future work should be dedicated to studying each of these factors, taking advantage of the strengths of different analog environments. With a more complete understanding, science objectives could be mapped to various potential ConOps, and future analog work could be focused on evaluating their effectiveness. For all ConOps, the design of EVA timelines that match the necessary tasks and level of required Earth–Mars interaction will be a key to their success.

BASALT project objectives do not include simulating rover operations or spacesuits. For BASALT field tests, traversing to and between science stations was simply conducted by walking and all EVA tasks were conducted shirtsleeve. On Mars, methods of traversing could include ambulating in a spacesuit, riding on an unpressurized rover in a spacesuit, or riding inside a pressurized rover; each of these options is associated with varying transport and EVA consumables costs and has advantages and limitations in regard to the types of terrain that can be crossed. On arrival at science locations of interest on Mars, some EVA operations will be conducted inside a spacesuit.

Hence, future work could incorporate (1) analog field testing that includes rover operations, (2) parallel suited testing using partial gravity offload systems with test subjects conducting relevant science tasks while wearing pressurized spacesuits and outfitted to capture relevant physiological parameters, and (3) modeling efforts that account for rover traverse capabilities (as opposed to shirtsleeve walking) and that extrapolate shirtsleeve field test data to that conducted in a pressurized suit. These efforts, along with complementary standalone technology research (*e.g.*, EV crew graphical display design, IV workstation layout, scientific field instrument design), could be combined to inform exploration EVA operations concepts, technology design requirements, and human health and performance drivers for EVA.

## 5. Conclusions

The BASALT project's Science Operations research conducted during BASALT-2 aimed at providing evidence-based recommendations for future planetary EVA. The overarching goal was to investigate the feasibility, value, and requirements for incorporating Earth-based scientific expertise within EVAs that were subject to Mars-relevant communication latencies and bandwidth limitations. All decisions regarding the design of EVA timelines and traverses, the definition of individual roles and responsibilities, and which capabilities to incorporate thoughtfully and deliberately intertwined science and science operations objectives.

The ConOps and capabilities tested during BASALT-2 incorporated precursor data-based plans with strategically designed EVA timelines that provided flexibility to react to data gathered by the EV crew in the field. The EV crew sequentially provided high-level context followed by detailed close-up information regarding candidate samples from three stations of scientific interest. This systematic, consecutive phasing of incoming information enabled the MSC to assimilate the incoming data and provide intra-EVA recommendations to the crew such that idle time was never incurred. The dynamic leaderboard approach provided a simple, yet powerful means by which the IV crew and MSC could track the most up-to-date science priorities.

The baseline architecture successfully implemented appropriate technologies to simulate Mars-forward capabilities to assist with communication between Mars-based EVA crew and an Earth-based MSC. These capabilities were evaluated under 5- and 15-min latencies and low and high bandwidth test conditions through a consensus-based ratings process that has been developed and validated through many previous analog missions. The high-level success of the baseline ConOps and objective data regarding BASALT-2 EVA execution has been presented here, whereas detailed presentation and discussion of the simulation quality, acceptability, and capability assessment ratings as well as answers to the Science Operations research questions are presented in the work of Beaton *et al.* (2019).

The ConOps and associated capabilities evaluated during BASALT-2 is one of the many approaches that could someday be implemented on Mars to facilitate scientific exploration and discovery. The knowledge gleaned from this analog test should be combined with the lessons learned from previous (and future) analogs to guide follow-on research objectives, implementations, and testing. Ultimately, these analog field tests should result in scientific, operational, and technological capabilities that will serve to inform the next generation of human-robotic planetary exploration.

## Abbreviations Used

BASALT: Biologic Analog Science Associated with Lava Terrains
CAPCOM: capsule communicator
ConOps: concepts of operations
DRATS: Desert Research And Technology Studies
EAMD: Exploration Analog and Mission Development
EV: extravehicular
EVA: extravehicular activity
EVIB: EV informatics backpack
FST: field support team
GAT: ground assimilation time
HST: Hawai‘i standard time
in-sim: in-simulation
ISS: International Space Station
IV: intravehicular
JSC: Johnson Space Center
LiDAR: light detection and ranging
MCC: Mission Control Center
MIP: mobile instrument platform
MMT: mission management team
MSC: Mission Support Center
NEEMO: NASA Extreme Environment Mission Operations
NLT: no-later-than
OWLT: one-way light time
PET: phase-elapsed time
PLRP: Pavilion Lake Research Project
RATS: Research And Technology Studies
SA: situational awareness
SCICOM: science communicator
SG: space-to-ground
SIMCOORD: simulation coordinator
xGDS: exploration ground data system
x-sim: out-of-simulation

## Notes

^*^ While idle time is not necessarily inherently detrimental, it has operational implications that need to be accounted for. For example, increased idle time increases overall EVA time, which is associated with added risk to the crew, additional requirements for consumables, etc.
